# The value of case reports

**DOI:** 10.3389/fvets.2025.1646659

**Published:** 2025-08-11

**Authors:** Koen M. Santifort, Rodrigo Gutierrez-Quintana, Natasha J. Olby, Björn P. Meij, Vicente Aige-Gil, Kaspar Matiasek, John H. Rossmeisl, Joao Miguel De Frias, Simone Spinillo, Bruno A. Lopes, Susana Monforte Monteiro, Richard A. LeCouteur, Martí Pumarola, Brian A. Summers

**Affiliations:** ^1^Department of Neurology and Neurosurgery, IVC Evidensia Small Animal Referral Hospital Arnhem, Arnhem, Netherlands; ^2^Department of Neurology and Neurosurgery, IVC Evidensia Small Animal Referral Hospital Hart van Brabant, Waalwijk, Netherlands; ^3^Department of Neurology and Neurosurgery, School of Biodiversity, One Health and Veterinary Medicine, College of Medicine, Veterinary Medicine and Life Sciences, University of Glasgow, Glasgow, United Kingdom; ^4^Department of Clinical Sciences, North Carolina State University, Raleigh, NC, United States; ^5^Department of Clinical Sciences, Faculty of Veterinary Medicine, Utrecht University, Utrecht, Netherlands; ^6^Department of Sanitat i Anatomia Animal, Faculty of Veterinary Medicine, Universitat Autònoma de Barcelona Campus UAB, Barcelona, Spain; ^7^Section of Clinical and Comparative Neuropathology, Institute of Veterinary Pathology, Centre for Clinical Veterinary Medicine, LMU Munich, Munich, Germany; ^8^Department of Small Animal Clinical Sciences, Virginia-Maryland College of Veterinary Medicine, Virginia Tech, Blacksburg, VA, United States; ^9^Hospital for Small Animals, Royal (Dick) School of Veterinary Studies, The University of Edinburgh, Edinburgh, United Kingdom; ^10^ChesterGates Veterinary Specialists, Chester, United Kingdom; ^11^Southfields Veterinary Specialists, Part of Linnaeus Veterinary Limited, Basildon, United Kingdom; ^12^Department of Veterinary Medicine, School of Biological Sciences, University of Cambridge, Cambridge, United Kingdom; ^13^School of Veterinary Medicine, University of California, Davis, Davis, CA, United States; ^14^Mouse and Comparative Pathology Unit, Department of Animal Medicine and Surgery, Faculty of Veterinary Medicine, Universitat Autònoma de Barcelona, Barcelona, Spain; ^15^Melbourne Veterinary School, University of Melbourne, Melbourne, VIC, Australia

**Keywords:** case reports, neurology, neurosurgery, neurophobia, education, development, learning, rare diseases

## 1 Introduction

The medical neurologists and neurosurgeons of the world constitute a huge professional body, focused on the disorders of the nervous system of a single species: humans. Subspecializations, such as in neonatal neurology, neuromuscular disorders, and geriatric neurology, have long evolved, given both the complexity of the conditions and the huge case numbers. Some neurological disorders (e.g., Alzheimer's and Parkinson's diseases) are of increasing importance globally. In striking contrast, veterinary neurology and neurosurgery is numerically a miniscule profession, yet tasked with investigating and treating conditions in several animal species, each with their own idiosyncrasies (cats are not small dogs). In many developed countries, there is now an expectation for sophisticated treatments for neurological disorders affecting beloved pets.

Veterinary neurology, like its counterpart in human medicine, often deals with rare, unexpected, complex, or poorly understood conditions. Moreover, the clinical presentations can be daunting for general practitioners and students, possibly leading to a fear of having to manage neurological patients (a phenomenon referred to as neurophobia in human and veterinary medical literature) (1–7). An extra factor contributing to this phenomenon in veterinary neurology, in comparison with its larger sister professions in humans, may be that scientific progress in veterinary neurology advances more slowly. Publications describe newly recognized disorders, superior imaging modalities, advances in neurosurgical procedures and improved therapeutic options, but case numbers are often small. Thus, while progress moves more slowly in the veterinary profession, case reports remain useful tools for documentation, education, and innovation. This article offers the collective opinion of 14 authors from the field of veterinary neurology on the contemporary value of case reports in veterinary neurology and neurosurgery. This paper resulted from the accumulated individual authors' opinions, which are provided in full in the Supplementary material file.

## 2 Collective opinion

Case reports (Box 1) offer an opportunity to challenge and advance what is known. They can be key to solving difficult and rare cases or form the basis of endeavors to explore new therapies and management approaches. Such reports will interest and aid those in general practice, more so specialists engaged in neurology. Novelty is often a factor of interest in these reports. However, not all case reports can, or need be novel and some will be justified if confirming or modifying findings in earlier communications, or describing the occurrence of syndromes in additional (different) animal breeds or global regions. Novelty is not the sole factor that comes into play when assessing the value of case reports. Even when similar diagnoses have been reported before, confirmation of earlier treatment results or rather contradictory outcomes can be of further value in the management of future cases. Surely, the conclusions in a few case reports may eventually prove to be erroneous but progress in medicine is always a work in progress.

Box 1 **Types of case reports.**

Case reports can be classified based on the number of cases presented, their pattern of occurrence, or their focus (e.g., treatment protocol). Types include:

- **Single case report**: describes a single patient's condition, diagnosis, treatment, and outcome. Often highlights a rare presentation or novel intervention. Specific formats, such as educational case reports or diagnostic challenge cases, are primarily used for teaching and clinical decision-making exercise.
- **Case report and literature review**: aside from information on a single case and discussion thereof based on the literature, a comprehensive overview of the field is provided.
- **Case series**: a descriptive study involving multiple cases (typically 2 to 5 for a small case series, 6 or more for a large case series) with shared characteristics.
- **Retrospective case series**: uses previously recorded cases from medical records.
- **Prospective case series**: identifies and follows cases as they occur in real time.
- **Cluster case report**: describes multiple similar cases that occur within a defined time period or geographic location, potentially indicating a common source, shared risk factor, or emerging condition.
- **Comparative case report**: compares two or more similar cases with different treatment strategies, outcomes, or diagnostic approaches.
- **N-of-1 (clinical) trial, pilot series, and first-in-man (or first-in-dog/cat/...)**: A type of single-patient trial using crossover comparisons (e.g., treatment vs. placebo) within the same individual. Designed to assess efficacy or tolerability in that specific patient and bridge the gap between clinical trials and individual care. First-in-man (or first-in-dog/cat/...) clinical case reports introduce a novel treatment in patients for which safety and efficacy was first tested in in vitro studies and/or experimental small animals (e.g., rodents).

Standardized formats like CARE guidelines (8) can increase consistency and quality of case reports.

Case reports introduce something new, and it is novelty that often inspires a case report to be written, which then opens the door to future research. Indeed, a novel patient that inspires a case report may trigger decades of investigation in its wake. For example, the first description of Alzheimer's Disease was a modest case report (9, 10). Few have such an exceptional and lasting impact. In veterinary neurology, a case report described the first case of “neurogenic lameness,” occurring in a pelvic limb due to a lateralized lumbar disc extrusion in a dog (11). This was important because it clarified a confusing clinical presentation. Another impactful case report led to the identification of *Neospora caninum* which hitherto had been masquerading as *Toxoplasma gondii*; nowadays, there is extensive literature about this protozoan and its impact in several species (12, 13). A series of reports on pituitary surgery in dogs and cats have offered a revolutionary way to treat various pituitary diseases and have broadened the scope of these disorders (14–16). The introduction of additive manufacturing (e.g., personalized 3D-implants) in veterinary medicine for complex orthopedic, neurologic, and oncologic cases are recently also documented through case reports (17).

Case reports can inform and reveal the necessary next step. They may explore and question the pathophysiology of diseases, raise potential toxicities, and warn about possible infectious disease outbreaks on the horizon. By presenting real-world clinical scenarios and challenges, they serve as excellent learning tools for students, practitioners, and researchers. They can illustrate clinical reasoning, disease management, and up-to-date techniques. Students and practitioners alike benefit from real clinical examples that reinforce diagnostic reasoning and elucidate anatomical-pathological correlations.

For many young veterinarians, writing a case report is their first experience in the world of scientific publishing. It offers valuable lessons in precisely documenting clinical observations and investigations, presenting a meaningful discussion of findings, and responding to peer review critiques.

For example, a case report describing a cat with myokymia and neuromyotonia was published in 2005 (18). Years later, one of the authors (NO) was contacted by a veterinarian about a cat with similar clinical signs. Treatment was guided by the previously published report and led to a positive outcome for the cat. While this does not constitute formal evidence for clinical decision-making, it nevertheless positively impacted the life of one cat—and that might make all the difference.

Specifically for the field of neuro-oncology and neurosurgery, case reports sometimes provide the best level of knowledge and can give new insights into the occurrence and diagnostic characteristics (including histopathological examinations) of certain tumor types. Recent case reports that documented unusual tumor types affecting the central nervous system or tumors affecting the central nervous system in remarkable patients (e.g., young age) often include diagnostic imaging study characteristics in addition to detailed clinical findings that help clinicians and pathologists to make the most out of such rare cases (19–21).

The language of case reports should reflect moderation and caution (22). With limited case numbers, broad generalizations are to be avoided but excessive reserve is unnecessary and might discourage further research. What is learned from a single case should not be generalized and conclusions drawn should recognize the limitations inherent in a single example. Gaps in knowledge should be clearly indicated. Much of the current knowledge in the field of veterinary neurology and neurosurgery relies on reports including small case numbers or observational studies that are inherently limited in determining causation or treatment efficacy. This is highlighted, for instance, by a recent review on the treatment of meningoencephalitis of unknown origin in dogs (23). Even when putting all the data of small case number-studies together, the ability to draw conclusions is limited. Indeed, the authors would encourage taking inspiration from case reports to collaborate in multi-center studies, especially in treatment case series or prospectively designed, randomized, blinded studies, in order to get a reasonable sample size allowing statistically meaningful analysis.

In academic assessment, case reports generally carry less weight than original research articles, which are based on more robust methodologies and include studies conducted under defined conditions with treatment and control groups. However, case reports can reflect sharp observational skills or the ability to interpret seemingly contradictory clinical findings, which lead to an important breakthrough in medical knowledge, or in hypothesis generation. The best of case reports are major contributions. A study in the human medical literature showed that about 25% of case reports from *The Lancet* led to subsequent larger studies, illustrating that case reports can play a crucial role in generating hypotheses that can lead to larger studies (24).

While case reports do not offer statistically significant proof in the way that large-scale studies or randomized controlled trials do, their value extends beyond mere statistical rigor. The argument that their low ranking in the evidence hierarchy diminishes their worth overlooks the critical role they play in medical knowledge and practice. Case reports sometimes serve as vital documentation for rare conditions that otherwise may be ignored in favor of major syndromes where a foundation of knowledge already exists. Case reports of the unusual can provide the breakthroughs that “get the ball rolling,” acting as catalysts for larger and more comprehensive studies guided by the initial findings. Additionally, they provide insights into unusual treatment responses, adverse effects, and complications that may not yet be broadly recognized. What can be viewed as a case report is the “N-of-1 clinical trial,” a type of single-patient study that uses crossover comparisons of different treatments (e.g., treatment vs. placebo) within the same individual. This type of trial may actually be one of the most reliable ways of determining the efficacy of treatment in that particular patient.

Similarly, first-in-man and first-in-dog/cat case reports or series can serve as vital links between “benchtop” and “bedside” studies. These reports are essential for establishing the safety and feasibility (though not efficacy) of novel treatments, particularly when placebo-controlled studies are not feasible—such as with the introduction of a new surgical technique. Even when extensive preliminary work has been conducted *in vitro, in silico, ex vivo* in cadavers, and *in vivo* in animal models (e.g., mice, rats, or rabbits), transitioning to patient studies remains a major leap; experimental animals with induced disease may not respond the same way as veterinary or human patients with naturally occurring disease. Ethically approved first-in-patient studies and case reports are therefore crucial early-stage contributions that pave the way for large-scale, prospective, randomized clinical trials aimed at evaluating efficacy and informing evidence-based practice. They can catalyze developments from benchtop to bedside. In this context, veterinary patients also play a unique role as translational animal models for spontaneous diseases shared between humans, dogs, and cats. This growing field, known as One Health–One Medicine, has generated numerous case reports that have benefitted both humans and their companion animals in meaningful, bidirectional ways (25–31).

The role of case reports as educational tools is also undeniable, both for the authors, who must use critical thinking and clinical reasoning skills, and for readers, who gain exposure to diagnostic and treatment challenges. Furthermore, in situations where more comprehensive studies are not feasible due to ethical or logistical constraints, case reports offer the best available evidence. As already stated, new diseases or syndromes often first emerge in the form of case reports with full comprehension only following additional interrogation. Perhaps most importantly, case reports inspire scientific curiosity by drawing attention to unique or unexpected cases; they encourage deeper inquiry and innovation. Their value, therefore, should not be judged solely on their ability to present a case but rather on their broader contributions to medical progress, education, and discovery.

Evaluating the value of a case report could benefit from a structured, quantitative approach. One example is the scoring system proposed by Pierson (32), although it is subject to variation between assessors. This method assesses reports based on their uniqueness, documentation quality, objectivity, interpretation, and educational value, each scored from 0 to 2, with a maximum of 10 points. A total score of 9–10 indicates a valuable contribution, 6–8 warrants cautious interpretation, and 5 or less suggests the report may not meet publication standards. Such frameworks support both authors and reviewers in assessing the potential impact and quality of a case report, fostering consistency and transparency in evaluating their merit.

The necessity of peer review of case reports has been questioned (33). Collectively, the authors of this opinion piece consider peer review to be of continued importance in case reports to verify the credibility of findings and conclusions. Reviewers play a crucial role in evaluating information and where needed, will request further investigations such as the examination of medical records or images or overlooked prior publications. Case reports have been compared with letters to the editor or website posts which can quickly disseminate clinical observations. However, these often lack rigorous peer review, and so again may be less credible and potentially ambiguous, leaving the reader to decide whether believable or not.

Peer review of case reports extends beyond validating scientific claims; it also supports the refinement of clinical storytelling and encourages a balanced discussion of (the impact of) errors, uncertainties, and decision-making under pressure. Given that case reports may describe scenarios where standard protocols were not feasible or applicable, such as lack of access to advanced diagnostic testing modalities, reviewers with broad clinical experience are well positioned to recognize both the challenges and the ingenuity involved. Their feedback can enhance the informative value of the report and ensure that the lessons drawn are appropriately contextualized. When formal studies are not feasible, thoughtful peer review can convert anecdotal insights into useful clinical knowledge.

In summary, the strengths and limitations of case reports (and ways to deal with them) are listed as follows:

### 2.1 Strengths of case reports

- Document unusual or new diseases, unexpected treatment responses, or novel complications.
  ° For rare diseases, they may provide the best available evidence of the presentation, clinical signs and course of action.
- Document the presence of known diseases in circumstances (e.g., geographical areas) previously not reported or thought exempt of disease (f.i. since a certain date) with legislative or One Health/zoonotic consequences (e.g., rabies).
- Inspire further research.
- Provide rich, in-depth descriptions of patient history, diagnostic workup, treatment course, and outcomes.
- Trigger clinical innovation by proof-of-concept.
- Describe safety and feasibility of novel treatments in first-in-man or first-in-dog/cat studies; serve as a translational link between “benchtop” and “bedside.”
- Serve as excellent learning tools.
- Highlight new therapeutic options.
- Are associated with low costs compared to formal studies.
- Can help detect and inform of new, rare, or late-onset adverse drug reactions (pharmacovigilance).
- Are useful when ethical constraints prevent experimental research, or are inevitable when translating novel treatments from the “benchtop” (*in vitro, in silico, ex vivo* cadaver studies) via *in vivo* experimental animals to the “bedside” (human and veterinary patients).
- Promote interdisciplinary collaboration which extends beyond patient care to a shared manuscript preparation.
- Can serve as sentinels for domestic family violence as reflected by animal abuse causing non-accidental injuries.
- Provide a realistic view of the clinical context, including decision-making challenges and socioeconomic considerations (factors involving social dynamics and financial constraints that may influence decision-making or outcomes) that are often underrepresented in research articles.
- Offer insights into methodological shortcomings, errors, or deviations from standard protocols such as the modality of imaging chosen or the medication administered that may have clinical or educational value.
  ° Consequences of errors that are discussed openly rather than concealed may help to prevent them in the future.
  ° Reasons for specific deviations from conventional protocols and the consequences may be illustrated.
- Enable reporting of cases from under-resourced areas with limited access to advanced diagnostics and treatment options. These may still yield valuable clinical insights.
- Help to collect, save and share observations, experiences, and conclusions gained from sporadic cases including different stages of the disease, together showing the evolution of a host-pathogen relationship.

### 2.2 Limitations (weaknesses) of case reports and ways to deal with them

- Findings may not be generalized and applied to a broader population due to the small sample size (often a single case).
  ° Compare findings with existing literature and similar cases (if such exist or follow subsequently) to identify patterns.
- An intervention followed by an outcome may suggest, but not necessarily establish, causation and deciding upon the appropriate and avoiding inappropriate therapy may be difficult.
  ° The patient may recover in spite of, rather than due to, the intervention.
  ° Clearly differentiate correlation from causation in the discussion and when interpreting the case report.
  ° Support findings with mechanistic explanations or pathophysiological reasoning.
  ° Study designs with control groups may follow to test initial suspected causation.
- Selection bias and reporting bias may lead to an overrepresentation of positive or unique cases.
  ° Acknowledge potential biases in case selection and reporting.
  ° Encourage publication of both positive and negative outcomes to balance the literature. Be honest.
- Small case reports lack statistical rigor and cannot establish definitive conclusions regarding some aspects of care such as treatment efficacy.
  ° Use case reports to generate scientific questioning and future study designs (e.g., cohort studies, experimental research).
  ° Use appropriate statistics when multiple cases are included.
- Readers may overgeneralize the findings.
  ° Emphasize what was learned from the case and provide clear limitations in the discussion—conclusions apply only to the small study group reported.
  ° Recommend further studies before applying findings broadly.
- Findings are difficult to validate or reproduce in controlled settings if only single cases are available.
  ° Include detailed methodology, diagnostic criteria, and treatment protocols to allow replication.
  ° Compare with similar reports to assess consistency.
- Limited availability of patient data (e.g., due to lack of access to comprehensive diagnostic investigations or lack of reporting thereof) or unclear reporting thereof may limit the conclusiveness of case reports and lead to recall bias.
  ° Check that all relevant available data of the particular case(s) is included.
  ° Use structured formats and timelines to prevent interpretive bias (bias caused by readers misinterpreting the information or drawing incorrect conclusions from the data or text).
  ° Use objective records (e.g., imaging, lab results) rather than relying solely on recall.
  ° Cross-check information with multiple sources, such as medical records and witness (owner) accounts.
- Focusing on the unusual might divert attention from common diseases or general interest.
  ° Keep the rare case in perspective: atypical presentation of known disorders is more common than something brand new. Discuss how the case contributes to broader clinical knowledge, such as differential diagnosis approaches.
  ° Avoid overemphasizing novelty when this is not the main point of the report, such as use of “this is the first…” type of sentences.

## 3 Discussion

In the context of veterinary neurology and neurosurgery, case reports are invaluable for documenting what is new, rare and novel, generating research questions, and providing educational insights; they often make for interesting reading. They inspire and propel the field forward. Despite limitations, they remain an essential component of medical literature when interpreted with appropriate caution and scholarly rigor. They offer unique opportunities for specialists to share their insights and contribute to the collective knowledge, ultimately benefiting animal health. They serve the One Health—One Medicine field for humans and animals. If not immediately, eventually they will be helpful in some circumstance, somewhere, sometime. Their value lies not in statistical significance, but in their power to inform, share invaluable clinical experience, and provoke thought and inquiry.

In conclusion, it is the collective opinion of the authors that while case reports have limitations, if these constraints are kept in mind, their contributions to clinical practice, education, and research are significant.

## References

[1] Abushouk AI Duc NM. Curing neurophobia in medical schools: evidence-based strategies. Med Educ Online. (2016) 21:32476. 10.3402/meo.v21.32476 27680578 PMC5040837

[2] Lambea-Gil A Saldaña-Inda I Lamíquiz-Moneo I Cisneros-Gimeno AI. Neurophobia among undergraduate medical students: a European experience beyond the Anglosphere. Rev Neurol. (2023) 76:351–9. 10.33588/rn.7611.2023102 37231548 PMC10478133

[3] Lin YW Volk HA Penderis J Tipold A Ehlers JP. Development of learning objectives for neurology in a veterinary curriculum: part I: undergraduates. BMC Vet Res. (2015) 11:2. 10.1186/s12917-014-0315-3 25582136 PMC4300725

[4] Lin YW Volk HA Penderis J Anderson TJ Añor S Lujan-Feliu-Pascual A. Development of learning objectives for neurology in a veterinary curriculum: part II: postgraduates. BMC Vet Res. (2015) 11:10. 10.1186/s12917-014-0314-4 25622644 PMC4323235

[5] Meyer FB Hoehne SN Murthy VD Maiolini A Stein VM Rathmann JMK. Perception of challenges in management of neurological cases in the emergency room. J Vet Emerg Crit Care. (2023) 33:38–46. 10.1111/vec.13258 36161761

[6] Murthy VD Le L Heater HD Guess SC Chen AV. Investigation of neurophobia amongst North American veterinary students and development of a veterinary neurophobia scoring tool (VetNeuroQ). J Vet Med Educ. (2024) 51:819–33. 10.3138/jvme-2023-0017 39504215

[7] Tarolli CG Józefowicz RF. Managing neurophobia: how can we meet the current and future needs of our students? Semin Neurol. (2018) 38:407–12. 10.1055/s-0038-1666987 30125894

[8] Riley DS Barber MS Kienle GS Aronson JK von Schoen-Angerer T Tugwell P. CARE guidelines for case reports: explanation and elaboration document. J Clin Epidemiol. (2017) 89:218–35. 10.1016/j.jclinepi.2017.04.026 28529185

[9] Alzheimer A. Über eine eigenartige Erkrankung der Hirnrinde. Allg Z Psychiatr Psych-Gerichtl Med. (1907) 64:146–8.

[10] Alzheimer A Stelzmann RA Schnitzlein HN Murtagh FR. An English translation of Alzheimer's 1907 paper, “Uber eine eigenartige Erkankung der Hirnrinde”. Clin Anat. (1995) 8:429–31. 10.1002/ca.980080612 8713166

[11] Bagley RS Pluhar GE Alexander JE. Lateral intervertebral disk extrusion causing lameness in a dog. J Am Vet Med Assoc. (1994) 205:181–5. 10.2460/javma.1994.205.02.181 7928570

[12] Bjerkås I Mohn SF Presthus J. Unidentified cyst-forming sporozoon causing encephalomyelitis and myositis in dogs. Z Parasitenkd. (1984) 70:271–4. 10.1007/BF00942230 6426185

[13] Dubey JP Carpenter JL Speer CA Topper MJ Uggla A. Newly recognized fatal protozoan disease of dogs. J Am Vet Med Assoc. (1988) 192:1269–85. 10.2460/javma.1988.192.09.1269 3391851

[14] van Blokland-Post K Grinwis GC Tellegen A Meij BP. Transsphenoidal hypophysectomy as a treatment for Rathke's cleft cyst in a dog. Vet Rec Case Rep. (2022) 10:e427. 10.1002/vrc2.427

[15] Meij BP van der Vlugt-Meijer RH van den Ingh TS Flik G Rijnberk A. Melanotroph pituitary adenoma in a cat with diabetes mellitus. Vet Pathol. (2005) 42:92–7. 10.1354/vp.42-1-92 15657280

[16] Meij BP van der Vlugt-Meijer RH van den Ingh TS Rijnberk A. Somatotroph and corticotroph pituitary adenoma (double adenoma) in a cat with diabetes mellitus and hyperadrenocorticism. J Comp Pathol. (2004) 130:209–15. 10.1016/j.jcpa.2003.09.004 15003481

[17] van den Brink EJC Grinwis GCM Willemsen K Driessen F Boroffka SAEB Meij BP. Additive manufacturing of titanium implants for skull reconstruction in 2 dogs after bone tumour excision. VCOT Open. (2023) 6:e61–6. 10.1055/s-0042-1758679

[18] Galano HR Olby NJ Howard JF Jr Shelton GD. Myokymia and neuromyotonia in a cat. J Am Vet Med Assoc. (2005) 227:1608–12, 1591. 10.2460/javma.2005.227.1608 16313038

[19] Gunovska H. Degl'Innocenti S, Targett M, Carrera I, Gomes SA. Case report: infiltrative multifocal meningioangiomatosis affecting the spinal cord of a young Labrador Retriever. Front Vet Sci. (2025) 12:1580306. 10.3389/fvets.2025.1580306 40557270 PMC12186452

[20] Ackerman LH Toonder M Bosch S Semenova VB Spicer TP Alas O. Multimodal treatment of a peripheral primitive neuroectodermal tumor originating from the thoracic cavity in a dog. J Vet Intern Med. (2025) 39:e70050. 10.1111/jvim.70050 40095239 PMC11912019

[21] Kristiansen KV Schrøder AS Bienzle D Vedel T Agerholm JS Berendt M. Lumbar round cell sarcoma in a 10-week-old rottweiler puppy. Acta Vet Scand. (2025) 67:12. 10.1186/s13028-025-00800-1 40075489 PMC11905653

[22] LeCouteur RA. The use of tentative language in scientific publications. J Vet Intern Med. (2024) 38:2980–1. 10.1111/jvim.17229 39431765 PMC11586534

[23] Jeffery N Granger N. New insights into the treatment of meningoencephalomyelitis of unknown origin since 2009: a review of 671 cases. Front Vet Sci. (2023) 10:1114798. 10.3389/fvets.2023.1114798 37008358 PMC10050685

[24] Albrecht J Meves A Bigby M. Case reports and case series from Lancet had significant impact on medical literature. J Clin Epidemiol. (2005) 58:1227–32. 10.1016/j.jclinepi.2005.04.003 16291466

[25] Dickinson PJ LeCouteur RA Higgins RJ Bringas JR Larson RF Yamashita Y. Canine spontaneous glioma: a translational model system for convection-enhanced delivery. Neuro Oncol. (2010) 12:928–40. 10.1093/neuonc/noq046 20488958 PMC2940703

[26] Destoumieux-Garzón D Mavingui P Boetsch G Boissier J Darriet F Duboz P. The one health concept: 10 years old and a long road ahead. Front Vet Sci. (2018) 5:14. 10.3389/fvets.2018.00014 29484301 PMC5816263

[27] Huenerfauth EI Bien CG Bien C Volk HA Meyerhoff N. Case report: Anti-GABA a receptor encephalitis in a dog. Front Vet Sci. (2022) 9:886711. 10.3389/fvets.2022.886711 35812851 PMC9262380

[28] Mwatondo A Rahman-Shepherd A Hollmann L Chiossi S Maina J Kurup KK. A global analysis of one health networks and the proliferation of one health collaborations. Lancet. (2023) 401:605–16. 10.1016/S0140-6736(22)01596-3 36682370

[29] Story BD Miller ME Bradbury AM Million ED Duan D Taghian T. Canine models of inherited musculoskeletal and neurodegenerative diseases. Front Vet Sci. (2020) 7:80. 10.3389/fvets.2020.00080 32219101 PMC7078110

[30] Vezza C Ruger L Langman M Vickers E Prada F Sukovich J. First-in-dog histotripsy for intracranial tumors trial: the FIDOHIST study. Technol Cancer Res Treat. (2024) 23:15330338241285158. 10.1177/15330338241285158

[31] Zinsstag J Schelling E Waltner-Toews D Tanner M. From “one medicine” to “one health” and systemic approaches to health and well-being. Prev Vet Med. (2011) 101:148–56. 10.1016/j.prevetmed.2010.07.003 20832879 PMC3145159

[32] Pierson DJ. How to read a case report (or teaching case of the month). Respir Care. (2009) 54:1372–8. 19796418

[33] Rishniw M. Do case reports warrant peer review? A critical analysis. Vet J. (2020) 262:105517. 10.1016/j.tvjl.2020.105517 32792092

