# Supplementary material for: The value of case reports
Source: Front Vet Sci. 2025 Aug 11;12:1646659. doi: 10.3389/fvets.2025.1646659 (PMC12375922; doi:10.3389/fvets.2025.1646659)
Supplement: Supplementary file 1 [file Data_Sheet_1.pdf]

frontiers

Supplementary Material

The opinion texts of each co-author are included as supplement to the opinion article in alphabetical order by surname.

João Miguel De Frias

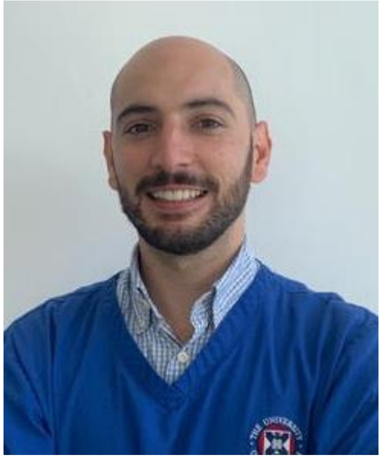

Case reports are often criticized for their inherent limitation of having a lack of significant impact on the scientific community. They are considered among the lower levels of the evidence hierarchy in science. Despite this, case reports are of undeniable importance. They present a unique opportunity to publish something novel. In fact, a large proportion of case reports in veterinary medicine include the word “first” in their abstract (1). However, case reports can be most impactful when generating hypotheses that lead to the creation of larger studies. A study indicated that about 25% of case reports and case series published in the high-impact medical journal The Lancet led to subsequent larger studies (2). Moreover, they offer a window for education by exploring and questioning the pathophysiology of certain diseases, identifying potential toxicities, and warning about possible infectious disease outbreaks.

Recently, because of concerns regarding reviewer fatigue and the low citation rates of case reports (which can lower journal impact scores), the idea of abolishing peer-review for case reports has been postulated (1). However, the process of peer-review is of utmost significance to verify the credibility of findings by experts in the field (3).

Considering the rarity described in case reports, the question arises: who would benefit most from them? Veterinary neurology practice is often found daunting by general practitioners and veterinary students. This is commonly referred to as neurophobia and is also observed in human medicine. To make neurology more approachable, strategies combining key concepts have been utilized, particularly clinical reasoning. Case reports challenge this approach by expanding what is known and predictable. Therefore, veterinary neurology case reports offer the opportunity to challenge and advance current knowledge. This particularly benefits specialists and clinicians working in the field. Novelty is often the key needed to solve a difficult case or to explore a new therapy or approach to a known condition.

This makes the case report a unique form of scientific writing, as it challenges established paradigms and fosters the generation of creative scientific questions. In my opinion, this is why peer-review remains of significant importance in case reports. Case reports cannot be replicated; they often describe singular rare or unique conditions, novel interventions, or novel diagnostic techniques. Reviewers have the crucial role of verifying information, either by drawing on personal experience to assess whether the authors’ claims are reasonable or, if possible, by examining their own data for similar outcomes. Moreover, reviewers should request medical records or images for verification when necessary. This validation not only gives credibility to the scientific report but also amplifies its spectrum of possible applications.

To conclude, while case reports are limited in nature, they offer an important opportunity—particularly for experts in the field—to challenge their *modus operandi* and generate new hypotheses for future research.

References:

1. Rishniw M. Do case reports warrant peer review? A critical analysis. Vet J. 2020 Aug;262:105517. doi: 10.1016/j.tvjl.2020.105517.
2. Albrecht J, Meves A, Bigby M. Case reports and case series from Lancet had significant impact on medical literature. J Clin Epidemiol. 2005 Dec;58(12):1227-32. doi: 10.1016/j.jclinepi.2005.04.003.
3. Gyles C. The medical case report. Can Vet J. 2017 Oct;58(10):1021-1023.

Vicente Aige-Gil

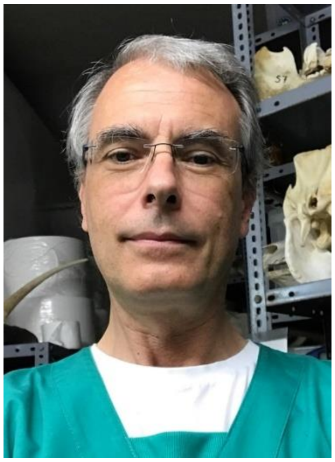

Anatomy is the study of form through dissection. The origin of the word *anatomy* comes from the Latin *anatomia* and the Greek *anatome*, with *ana-* meaning "up" or "apart" (implying division) and -*tome* meaning "cutting." Dissection is the fundamental tool and basis of anatomical study.
Etymologically, *dissection* originates from the Latin *dissecare* ("dis-" meaning "apart" and "secare" meaning "to cut"), describing the practice of studying anatomy by methodically cutting and separating bodily structures.
The role of the anatomist is to study cadavers and live specimens, comparing individuals and species while recognizing that variations are a natural part of biology. However, form and function are interdependent, as function always responds to form. This is why anatomists must seek explanations to better understand structure. This is a particularly important aspect in both teaching and research.

Building on this foundation, the next step for an anatomist is to engage with clinical cases and contribute to the morphological analysis of pathological conditions. In this context, case-related publications serve as educational tools, disseminating knowledge about specific pathological conditions not only to clinicians but also to anatomists themselves. This is why clinicians and anatomists should work hand in hand to study and analyze cases. The clinician will learn from the anatomist, and vice versa.

When reporting images obtained with specific diagnostic tools, anatomical knowledge becomes a cornerstone for identifying and localizing lesions and for understanding their implications. An imaging specialist must have a very strong understanding of anatomy.

When addressing a case report, the description of the normal anatomy should precede the description of the anomaly. This approach facilitates the understanding of the pathological condition, aids in reaching a correct diagnosis, and assists in selecting the appropriate therapeutic option. Understanding normal form and function is essential to understanding malfunction.

Therefore, clinical case report publications are excellent tools not only for spreading knowledge among clinicians but also for raising awareness of the critical role that normal anatomy plays in diagnosing and understanding pathological conditions.

Rodrigo Gutierrez-Quintana

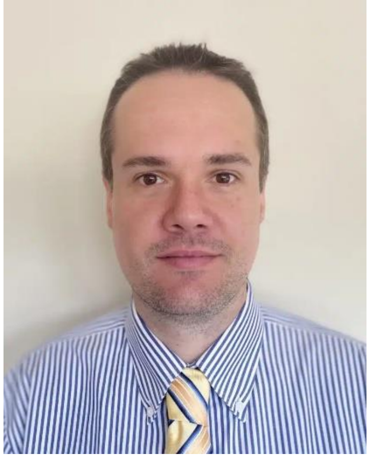

Case reports are descriptive studies that typically present a detailed account of the diagnosis, treatment, and follow-up of an individual patient. They are popular and have a long-standing tradition in the veterinary literature. Although they have one of the lowest rankings on the evidence hierarchy, their contribution to clinical practice, education, and research should not be underestimated.

Strengths of case reports

- **Detecting novelties:** Case reports are the only way to quickly report novel or unusual, uncontrolled observations regarding clinical signs, clinical findings, treatments, or outcomes. They are often the first to document new diseases.
- **Rare diseases:** Research on rare diseases may need to rely on small case series or case reports due to the limited number of patients available for recruitment.
- **Generating hypotheses:** Case reports contribute to the generation of new research questions that can later be explored in larger studies. There are many examples of major discoveries that began as case reports.
- **Pharmacovigilance:** Case reports act as a primary source of information for detecting new, rare, or late-onset adverse drug reactions, particularly those not identified during clinical trials.
- **Solving ethical constraints:** Case reports can be valuable when ethical constraints prevent experimental research. For example, they provide insight into managing clinical complications or side effects that would not be ethical to induce experimentally.
- **Detailed description:** Case reports tend to present clinical findings in greater detail than larger studies.
- **Educational value:** Case reports illustrate day-to-day clinical practice, clinician diagnostic reasoning, disease management, and follow-up information. They are valuable for teaching the best approaches to clinical cases and for keeping up to date with advanced techniques and treatments. They also serve as an entry point for young researchers and clinicians to contribute to the scientific literature.
- **Lower expenses:** The cost of producing a case report is low compared to planned, formal studies, as most of the necessary work is typically carried out during routine clinical practice without specific funding.

Weaknesses and limitations of case reports

- **Lack of generalizability:** Findings from a case report cannot be generalized.
- **No possibility to establish cause-effect relationships:** Causality cannot be inferred from an uncontrolled observation. An association does not imply a cause-effect relationship.
- **Risk of overinterpretation:** There is a tendency or temptation to generalize findings without adequate justification.
- **Publication bias:** Authors and journals generally favor the publication of positive-outcome findings.

- **Retrospective design:** Medical records may not contain all relevant data, and there may be recall bias.
- **Focus on the unusual:** Because case reports often deal with rare or atypical presentations, they may inadvertently divert attention from common diseases and presentations.

In conclusion, while case reports have limitations due to their inherent design, they remain a valuable tool in veterinary neurology research—particularly for identifying novel findings, generating hypotheses, and contributing to medical education, especially in the context of rare diseases or unusual clinical scenarios. However, it is important to interpret them with caution and to recognize their limitations. Consistency and quality could be improved by using standardized formats such as CARE (Case Report Guidelines).

References:

1. Lowenfels AB, Mamtani R, Solomon LW, Maisonneuve P, Cheema S. The Value of Case Reports for Graduate Medical Education. J Grad Med Educ. 2022 Oct;14(5):529-532. doi: 10.4300/JGME-D-21-01115.1.
2. Nissen T, Wynn R. The clinical case report: a review of its merits and limitations. BMC Res Notes. 2014 Apr 23;7:264. doi: 10.1186/1756-0500-7-264.
3. Elliott JEG. The value of case reports in diagnostic radiography. Radiography (Lond). 2023 Mar;29(2):416-420. doi: 10.1016/j.radi.2023.01.028.
4. Garcia-Doval I, Segovia E, Hunter H, Frew J, Naldi L. The value of case reports in pharmacovigilance. Br J Dermatol. 2020 Nov;183(5):795-796. doi: 10.1111/bjd.19504.
5. Riley DS, Barber MS, Kienle GS, Aronson JK, von Schoen-Angerer T, Tugwell P, Kiene H, Helfand M, Altman DG, Sox H, Werthmann PG, Moher D, Rison RA, Shamseer L, Koch CA, Sun GH, Hanaway P, Sudak NL, Kaszkin-Bettag M, Carpenter JE, Gagnier JJ. CARE guidelines for case reports: explanation and elaboration document. J Clin Epidemiol. 2017 Sep;89:218-235. doi: 10.1016/j.jclinepi.2017.04.026.

Richard A. LeCouteur

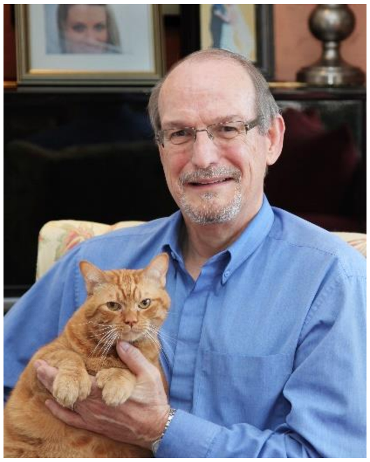

Single case reports are valuable contributions to the veterinary medical literature, offering detailed insights into rare conditions, novel treatments, or unexpected clinical presentations. However, their limitations must be acknowledged to ensure proper interpretation and application.

Strengths

- Case reports may document unusual diseases, unexpected treatment responses, or novel complications that are not yet represented in the literature.
- They can inspire further research by identifying potential risk factors, treatment effects, or diagnostic challenges that warrant controlled studies.
- They provide rich, in-depth descriptions of patient history, diagnostic workup, treatment course, and outcomes.
- They serve as excellent learning tools for students, practitioners, and researchers by presenting real-world clinical cases and challenges.
- Some of the first reports of new diseases or syndromes originated as case reports, highlighting their importance in recognizing emerging health threats.
- For rare diseases where clinical trials are impractical, case reports provide some of the best available evidence.

Limitations

- Since findings are based on a single patient or a small number of cases, they may not apply to a broader population.
- Without a comparative element (e.g., a control group), it is difficult to establish causation between interventions and outcomes.
- Selection bias and reporting bias may lead to an overrepresentation of positive or unique cases, skewing clinical perceptions.
- Case reports lack statistical rigor and cannot establish definitive conclusions about disease processes or treatments.
- Readers may overgeneralize the findings, mistakenly assuming that the observed outcome will be reproducible in other cases.
- As they rely on anecdotal evidence, findings from case reports are difficult to validate or reproduce in controlled settings.

Case reports vs. letters to the editor or website posts
A letter to the editor or a website post may serve a similar function to a case report by sharing unique clinical experiences. However, there are notable differences in impact and scientific rigor.

Advantages of letters or website posts compared to case reports

- Allow for quick dissemination of clinical observations.
- Permit a more concise discussion without the structured approach of a formal case report.
- Provide a platform for clinicians to discuss findings and exchange ideas.

Disadvantages of letters or website posts compared to case reports

- Most letters and website posts do not undergo rigorous peer review, making them less credible.
- Space constraints may prevent comprehensive analysis and discussion.
- Unlike case reports, they are not indexed in major research databases.
- Website posts may not be archived, reducing their long-term impact.

Conclusion

Single case reports are valuable, offering insights into rare conditions and serving as a basis for hypothesis generation. However, case reports should be interpreted cautiously due to their inherent limitations. Proper distinction between case reports and original research is essential in academic assessment, ensuring that contributions are appropriately weighted in CV evaluations and professional recognition. Balancing tentative language (e.g., “probable,” “possible,” “presumed”) with meaningful clinical insights allows case reports to serve their intended purpose without overstating their significance.

Bruno Lopes

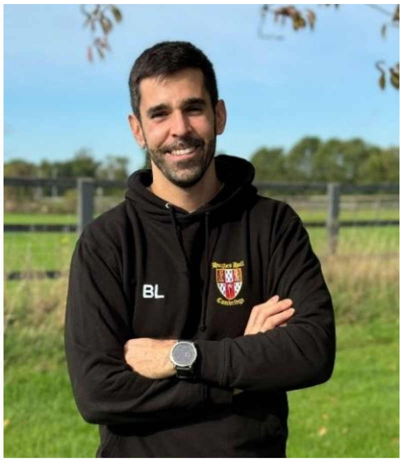

Case reports are an important and valuable part of the medical literature. Being short publications, they allow clinicians to thoroughly describe and share particular cases or presentations with the community. Case reports generally fall quite low in the hierarchy of scientific literature. They have pros and cons and should be used under specific conditions. However, they are a unique way to complement the scientific literature and advance our knowledge.

One of the most significant contributions of case reports is their ability to document rare or novel medical conditions.
By documenting these cases, they contribute to a collective knowledge base that can aid others in diagnosing and treating similar conditions or in being aware of unusual presentations of known diseases.

Additionally, case reports often serve as the foundation for future research. There are numerous examples where an initial short communication of an atypical case presentation led to further curiosity, sharing of cases, and eventually finding the underlying cause. A good example (among many others) includes the first identification of *Neospora caninum* by Bjerkås, Mohn, and Presthus (1984), which uncovered a new cyst-forming protozoan that was eventually characterized by Dubey et al. (1988) (1,2). Nowadays, there is extensive literature about this protozoan and its impact on several species.

Finally, case reports also have educational value. They can be used as invaluable teaching tools, presenting real-world clinical scenarios and their associated challenges to the broader community. Furthermore, a case report often represents the first contact that young veterinarians in training have with scientific publication. More often than not, young interns wishing to challenge themselves and further their knowledge propose writing a case report, generally under the guidance of a senior clinician. This exposes young veterinarians to thorough investigations aimed at reaching a correct diagnosis, and to the challenges of scientific writing and peer review. This is an important experience prior to undertaking larger projects that rank higher in the hierarchy of scientific literature.

However, care should be taken when considering writing a case report. Often, in the urge to find a publishable case, there is an attempt to report cases that are either not truly unique or not well documented. On one hand, this may lead to an excess of case reports that become lost in the vast amount of scientific literature available; on the other hand, it may lead to frustration if peer reviewers do not find the proposed manuscript relevant. Occasionally, it happens that the case being described is not that uncommon; in such instances, there should be an effort by the veterinary community to come together and produce a more comprehensive study.

In summary, case reports remain an invaluable component of the veterinary literature, offering the opportunity to report unique or novel cases. Nonetheless, it is important to review the existing literature to understand the relevance of the case at hand and ensure that adequate investigations are conducted to provide a meaningful addition to the literature.

References:

1. Bjerkås I, Mohn SF, Presthus J. Unidentified cyst-forming sporozoon causing encephalomyelitis and myositis in dogs. Z Parasitenkd. 1984;70(2):271-4. doi: 10.1007/BF00942230.
2. Dubey JP, Carpenter JL, Speer CA, Topper MJ, Uggla A. Newly recognized fatal protozoan disease of dogs. J Am Vet Med Assoc. 1988 May 1;192(9):1269-85.

Kaspar Matiasek

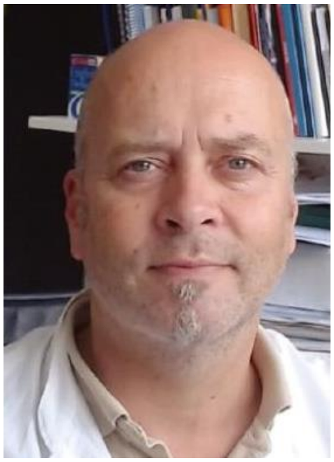

Challenges of good case reporting

The illustration of clinical cases can provide a fantastic window into the clinical field and the story behind novel encounters, provided they comply with defined quality standards.

Diagnosticians may benefit in particular from formative documentation in an algorithmic order, while those seeking treatment solutions may gain essential clues from sophisticated clinical reasoning and proper follow-up. On the other hand, readers also learn from the psychosocial and economic struggles, methodological shortcomings, mishaps, errors, and misinterpretations—narratives often omitted from scientific contributions but, even in the most renowned institutions, part of daily practice.

The advancement of evidence-based medicine and consensus statements by peer veterinarians in the field and their medical counterparts now provide a fairly reliable framework for the work-up, management, and monitoring of neurological patients. Consequently, it is the responsibility of clinicians to follow these guidelines and of authors to document compliance and explain any chosen alternatives. On a global scale, the geographic distribution of specialist knowledge and the availability of diagnostic technologies (or the funding to run them) pose major challenges to the scientific community. Consultation on a broad basis may help with the work-up remotely, but the lack of "technical" data (laboratory, diagnostic imaging, gene testing, etc.) and treatment options will continue to impede case reports from less well-equipped areas. We may therefore miss interesting entities tied to certain environments and local animal populations.

Apart from proper work-up and documentation, the case being reported must be unique, a rare or even unknown entity, or at least unusual in its presentation and clinical context. While evaluating the report's style and strength of data may appear straightforward, recommendations for clinical reasoning and management of the unique case may be lacking, leaving the reviewer with their own interpretation. It is definitely advantageous if the reviewers have seen and struggled with a larger number of cases themselves, as this experience will help them provide valuable assistance in improving the report's quality rather than simply judging it.

A helpful scheme for scoring the value of case reports was proposed by Pierson (1), who implemented a system that evaluates their uniqueness/originality, documentation strength, interpretation versus objectivity, and educational value. This scoring system uses a scale from 0–2 for each determinant, with a maximum total score of 10. A total score of 9–10 indicates that the report is a worthwhile contribution to the literature. A score of 6–8 suggests that the reader should be cautious about its validity and clinical value. A score of 5 or less suggests that the report should be rejected.

Ultimately, the curriculum leading to board eligibility in neurology should equip clinicians with sufficient clinical reasoning and the attitude required to work up unusual cases to high standards and to seek assistance from other experts and literature when needed. Case reports are an excellent format to reflect the clinical situation, with its intimidating responsibilities and the need for ad-hoc responses that can lead to long-lasting consequences. There is no better way to learn than from individual and true clinical experiences, even if made by others. New case reports may help, retrospectively, to solve

“cold” cases if they have been worked up and reported according to appropriate standards. It is the responsibility of experts in the field to support this process by being available as reviewers, whose criticism and recommendations provide essential tools not only to improve the report but also to help improve the clinical work-up of future patients.

References:

1. Pierson DJ. How to read a case report (or teaching case of the month). Respir Care. 2009 Oct;54(10):1372-8.

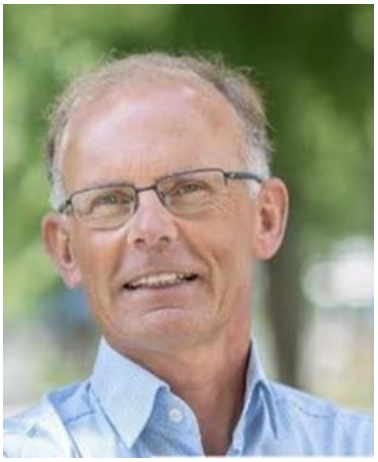

Björn P. Meij

Case reports play a crucial role in advancing veterinary medicine. They serve as detailed accounts of individual animal cases, highlighting unique clinical presentations, diagnostic challenges, treatment approaches, and outcomes. Case reports also contribute to research areas, stimulate knowledge sharing, and may initiate new collaborations. This format provides invaluable insights that can inform both clinical practice and future research.

1. Identification of rare diseases and changes in the paradigm of treatment

Veterinary medicine encompasses a wide range of species and conditions. Case reports can identify rare diseases or unusual presentations of common conditions. For instance, when hypophysectomy was introduced as a treatment for dogs and cats with Cushing's disease at Utrecht University (the Netherlands) in 1993 (1–3), it became an established treatment for dogs (4). During this period, we encountered and confirmed a range of new pituitary diseases, apart from the classical corticotroph cell adenoma that causes Cushing's disease (5). Our group described each of these pituitary entities separately in case reports, such as Rathke's cleft cyst (6), melanotroph adenoma (7), and double pituitary adenoma (8). The value of case reports is not only to document rare cases but also to show that, among the typical presentations of dogs with pituitary masses, there may be diseases—like lymphocytic hypophysitis—that are not indications for surgical treatment (9). Another example is the discovery of ectopic secretion of adrenocorticotropic hormone (ACTH) in a case that presented with ACTH-dependent hypercortisolism and underwent hypophysectomy, which did not resolve the clinical signs (10). Thus, we learned the hard way by performing hypophysectomy in cases that had non-neoplastic (9) or extra-pituitary (10) disease. These case reports illustrate previously unknown pathogenesis and pathophysiology of well-known clinical syndromes, such as diabetes insipidus and Cushing's syndrome.

Another example is the case documentation in 2010 of a cat with insulin-resistant diabetes mellitus (DM) due to a growth hormone (somatotroph) pituitary adenoma that underwent hypophysectomy with an excellent clinical outcome, reflected in full remission of diabetes mellitus (11). A decade later (2021), two groups (UK and the Netherlands) reported similar findings in a large cohort of cats (12,13), and hypophysectomy is now established as the treatment of choice for hypersomatotropism due to somatotroph pituitary adenoma in cats with insulin-resistant DM.

Thus, case reports on pituitary surgery in dogs and cats over the last three decades have contributed to greater awareness of various pituitary diseases, new approaches to pituitary disease, and improved diagnostic techniques among veterinarians, enhancing animal welfare through timely interventions. They also contributed to changing the paradigm in veterinary medicine from medical treatment to pituitary surgery (14,15).

2. Foundation for research

Case reports often serve as the basis for larger studies (11,12,13). They can reveal trends, generate hypotheses, and identify areas requiring further investigation. Another example showing the value of case reports in initiating a new research area is the introduction and application of additive manufacturing (AM, 3D printing) in veterinary medicine. The use of customized implants has

enabled personalized treatment for complex cases with oncologic and orthopedic diseases manifesting as large bone defects. Previously, there were no other treatments available for these patients other than palliative care or amputation of a limb. Since the introduction of AM in the veterinary field, new treatments have become possible, leading to complete cures, and these were initially described in case reports, such as in dogs with large skull bone tumors (16) or critically sized bone defects of the antebrachium (17). These case experiences led to the development of a novel surgical technique, shelf arthroplasty (3DHIP), for a common disease like hip dysplasia in young dogs with hip laxity (18). Previously, this condition was treated with invasive double/triple pelvic osteotomy. In a relatively short time, 3DHIP became the treatment of choice in a large cohort of dogs (19), and ongoing clinical trials showed that this procedure is a favorable alternative to invasive pelvic osteotomies. Translational research, performed in collaboration with human orthopedic surgeons seeking a less invasive surgical alternative to complicated acetabular osteotomies for young adults with hip dysplasia, is ongoing (20). Investigation of long-term clinical results in dogs treated with 3DHIP is important for the potential application in human counterparts.

3. Educational resource and knowledge sharing

Case reports are particularly beneficial for veterinary students and practitioners. They illustrate real-life scenarios, enhancing learning by contextualizing theoretical knowledge. For example, a detailed case report can guide veterinarians in recognizing clinical signs and implementing effective diagnostic protocols. This hands-on learning approach often complements traditional textbooks and lectures. The dissemination of case reports through journals and conferences fosters collaboration among veterinarians. Sharing experiences and outcomes encourages a culture of learning and innovation. For example, our published case report (11) on a novel treatment protocol for somatotroph pituitary adenoma prompted others (13) to adopt a similar approach, but also led to new collaborations and referrals across borders (12), ultimately improving patient outcomes.

In summary, case reports are invaluable in veterinary medicine, offering educational benefits, aiding in disease recognition, facilitating knowledge sharing, and laying the groundwork for future research. Their contribution is essential to the continuous improvement of veterinary practice and animal health.

References:

1. Meij BP, Voorhout G, Van den Ingh TS, Hazewinkel HA, Van't Verlaat JW. Transsphenoidal hypophysectomy in beagle dogs: evaluation of a microsurgical technique. Vet Surg. 1997 Jul-Aug;26(4):295-309. doi: 10.1111/j.1532-950x.1997.tb01502.x.
2. Meij BP, Voorhout G, van den Ingh TS, Hazewinkel HA, Teske E, Rijnberk A. Results of transsphenoidal hypophysectomy in 52 dogs with pituitary-dependent hyperadrenocorticism. Vet Surg. 1998 May-Jun;27(3):246-61. doi: 10.1111/j.1532-950x.1998.tb00123.x.
3. Meij BP, Voorhout G, Van Den Ingh TS, Rijnberk A. Transsphenoidal hypophysectomy for treatment of pituitary-dependent hyperadrenocorticism in 7 cats. Vet Surg. 2001 Jan-Feb;30(1):72-86. doi: 10.1053/jvet.2001.17843.
4. van Rijn SJ, Galac S, Tryfonidou MA, Hesselink JW, Penning LC, Kooistra HS, Meij BP. The Influence of pituitary size on outcome after transsphenoidal hypophysectomy in a large cohort of dogs with pituitary-dependent hypercortisolism. J Vet Intern Med. 2016 Jul;30(4):989-95. doi: 10.1111/jvim.14367.
5. Sanders K, Galac S, Meij BP. Pituitary tumour types in dogs and cats. Vet J. 2021;270:105623. doi: 10.1016/j.tvjl.2021.105623.

6. van Blokland-Post K, Grinwis GC, Tellegen A, Meij BP. Transsphenoidal hypophysectomy as a treatment for Rathke's cleft cyst in a dog. Vet Rec Case Rep. 2022;10:e427. doi.org/10.1002/vrc2.427
7. Meij BP, van der Vlugt-Meijer RH, van den Ingh TS, Flik G, Rijnberk A. Melanotroph pituitary adenoma in a cat with diabetes mellitus. Vet Pathol. 2005 Jan;42(1):92-7. doi: 10.1354/vp.42-1-92.
8. Meij BP, van der Vlugt-Meijer RH, van den Ingh TS, Rijnberk A. Somatotroph and corticotroph pituitary adenoma (double adenoma) in a cat with diabetes mellitus and hyperadrenocorticism. J Comp Pathol. 2004 Feb-Apr;130(2-3):209-15. doi: 10.1016/j.jcpa.2003.09.004.
9. Meij BP, Voorhout G, Gerritsen RJ, Grinwis GC, Ijzer J. Lymphocytic hypophysitis in a dog with diabetes insipidus. J Comp Pathol. 2012 Nov;147(4):503-7. doi: 10.1016/j.jcpa.2012.04.006.
10. Galac S, Kooistra HS, Voorhout G, van den Ingh TS, Mol JA, van den Berg G, Meij BP. Hyperadrenocorticism in a dog due to ectopic secretion of adrenocorticotropic hormone. Domest Anim Endocrinol. 2005 Apr;28(3):338-48. doi: 10.1016/j.domaniend.2004.11.001.
11. Meij BP, Auriemma E, Grinwis GC, Buijtels JJ, Kooistra HS. Successful treatment of acromegaly in a diabetic cat with transsphenoidal hypophysectomy. J Feline Med Surg. 2010 May;12(5):406-10. doi: 10.1016/j.jfms.2010.03.014.
12. van Bokhorst KL, Galac S, Kooistra HS, Valtolina C, Fracassi F, Rosenberg D, Meij BP. Evaluation of hypophysectomy for treatment of hypersomatotropism in 25 cats. J Vet Intern Med. 2021 Mar;35(2):834-842. doi: 10.1111/jvim.16047.
13. Fenn J, Kenny PJ, Scudder CJ, Hazuchova K, Gostelow R, Fowkes RC, Forcada Y, Church DB, Niessen SJM. Efficacy of hypophysectomy for the treatment of hypersomatotropism-induced diabetes mellitus in 68 cats. J Vet Intern Med. 2021 Mar;35(2):823-833. doi: 10.1111/jvim.16080.
14. Rivenburg R, Owen TJ, Martin LG, Chen AV. Pituitary surgery: changing the paradigm in veterinary medicine in the United States. J Am Anim Hosp Assoc. 2021 Mar 1;57(2):73-80. doi: 10.5326/JAAHA-MS-7009.
15. Meij BP, van Stee LL. Transsphenoidal surgery for pituitary tumors. Vet Clin North Am Small Anim Pract. 2025 Jan;55(1):95-118. doi: 10.1016/j.cvsm.2024.07.009.
16. van den Brink EJC, Grinwis GCM, Willemsen K, Driessen F, Boroffka SAEB, Meij BP. Additive manufacturing of titanium implants for skull reconstruction in 2 dogs after bone tumour excision. VCOT Open 2023;6:e61–e66. doi.org/10.1055/s-0042-1758679
17. Janssens SDS, Willemsen K, Magré J, Meij BP. Additive titanium manufacturing to repair critically sized antebrachial bone defects in two dogs. VCOT Open. 2023;6:e75–e83. doi.org/10.1055/s-0043-1769011
18. Kwananocha I, Verseijden F, Kamali SA, Magré J, Willemsen K, Schouten JC, Salvatori D, Tryfonidou MA, Meij BP. Surgical technique of the 3-dimensional printed personalized hip implant for the treatment of canine hip dysplasia. J Vis Exp. 2024;206:e66005. doi:10.3791/66005.
19. Kwananocha I, Magré J, Kamali A, Verseijden F, Willemsen K, Ji Y, van der Wal BCH, Sakkers RJB, Tryfonidou MA, Meij BP. Outcome one year after acetabular rim extension using a customized titanium implant for treating hip dysplasia in dogs. Animals. 2024;14:2385. doi.org/10.3390/ani14162385

20. Willemsen K, Möring MM, Harlianto NI, Tryfonidou MA, van der Wal BCH, Weinans H, Meij BP, Sakkers RJB. Comparing hip dysplasia in dogs and humans: a review. Front Vet Sci 2021;8:791434. doi: 10.3389/fvets.2021.791434

Susana Monforte-Monteiro

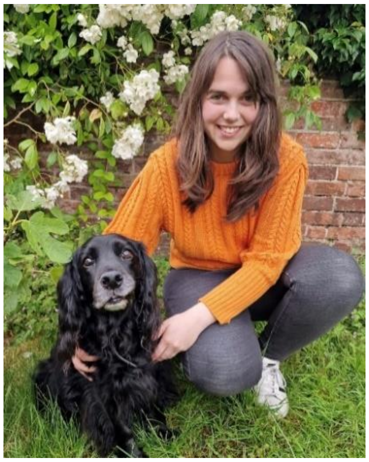

Often, in everyday practice, veterinary clinicians encounter interesting cases. These cases can be considered interesting for a multitude of reasons: an unusual presentation of a well-documented disease, a new finding in a diagnostic test for a certain condition, or a treatment that had been attempted for similar disorders but proves successful in a different disease. Writing and publishing a report on these findings is often the first step in the advancement of the medical profession. Several factors contribute to this:

- The clinician-scientist will review the pathophysiology of the disease and the current available literature, explaining why the case is important to share. Hypotheses that could justify the findings are often generated at this point.
- There is an increased awareness of the different presentations, diagnostics, or treatments for a particular condition that could help several individual patients in the long term once shared with colleagues in the veterinary field.
- Sharing the information could lead to large prospective studies relying on observational and experimental research methods, which could further advance medical knowledge.

The importance of case reports is well recognized in human medicine. Sir William Osler, a well-known physician and founding professor of Johns Hopkins Hospital, once said: "Always note and record the unusual… Publish it. Save it on a permanent record as a short, concise note. Such communications are always of value." (1). It is also not unusual to find more than one case report of the same uncommon finding or even disease process in human medicine. Once a few reports have been published, these are collated, and a case series along with a review of the literature is made available—this makes the quest for information about any such condition much easier for practitioners. A good example of the importance of reporting unusual signs can be seen in the initial description of Parkinson's disease by John Parkinson as a case series in 1817 (2).

An additional benefit of case reports and case series comes from providing evidence from naturally occurring situations for which experimental studies cannot be designed (e.g., experiments that would be harmful to the individuals).

However, care should be taken when interpreting the information provided by case reports. No cause-effect relationship can be inferred from case reports, and the lack of a representative population makes generalization impossible, although case series can mitigate this problem somewhat. A positive bias is also common among case reports. This is particularly evident when reporting a new treatment, with the majority describing only positive outcomes. Case reports documenting negative outcomes contribute equally to the medical literature and should be considered for publication.

Case reports and case series are an invaluable part of medical and veterinary literature, and their contribution—often as the first step in advancing veterinary medical knowledge—should not be underestimated.

References:

1. Thayer WS. Osler, the teacher sir William Osler, Bart. Baltimore: Johns Hopkins Press; 1920:51-2.
2. Parkinson J 1817. An essay on the shaking palsy. Whittingham and Rowland for Sherwood, Needly and Jones, London

Natasha J. Olby

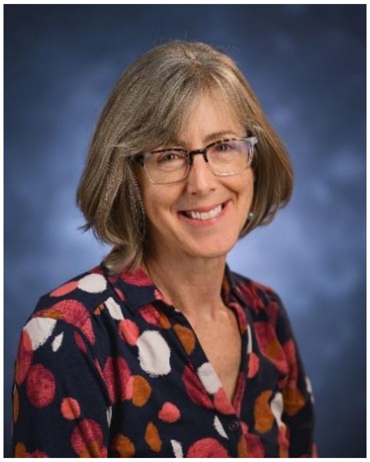

There is enormous value (and joy) in discussing interesting cases with colleagues. The exchange of anecdotes about novel presentations, unexpected findings, and interesting treatments provides a route to new ideas and challenges entrenched views. Case reports represent the published version of this exchange, presented in a more formalized manner with careful documentation of what is often (but not always) a rare phenomenon or a novel approach. These publications are not intended to provide scientific evidence for a mechanism of disease or therapy, but they can alert the community to something new and set the stage for future studies.

Perhaps one of the best examples of the power of a case report is the first description of Alzheimer's Disease, written in 1907 by Alois Alzheimer (1). This carefully written paper (a translation is now available (2)) described the clinical signs and histopathology of a 51-year-old woman suffering from dementia. As we all know, this disease is not so rare, and his extremely detailed and accurate descriptions and conclusions formed the basis of all future work on Alzheimer's Disease.

In the world of veterinary neurology, I reference a case report on intervertebral disc disease by Bagley et al., published in 1994 (3). It described a Cocker Spaniel presenting with right hind limb lameness due to a lateralized disc extrusion. This was the first description of this phenomenon in the lumbar (rather than cervical) spine, highlighting the tunnel vision we can develop as we distinguish orthopedic from neurologic disease. It was a well-written summary of a relatively unusual presentation that can easily be missed, particularly in the days before cross-sectional imaging was commonly used. For this case report, the description was followed by a discussion from two different specialists, which reflected the value of case reports in triggering discussion and the exchange of ideas.

In some instances, a case report can be life-changing for an individual animal. When clinicians are faced with a challenging or unusual case, finding a comparable description in a case report allows them to validate their findings and explore treatment options and potential outcomes. This was emphasized for me recently. In 2005, we published details of a very unusual cat that presented with rhythmic muscle movements and progressive contractures of its carpi, making walking difficult (4). Ultimately, we diagnosed the cat with myokymia and neuromyotonia, and it responded to carefully dosed phenytoin. We collaborated with Diane Shelton and a medical neurologist (James Howard) to reach the diagnosis. It was a strange enough case, with a good outcome, that we decided to share it with colleagues. Imagine my surprise when, over 20 years later, I was contacted by a cat owner whose cat was exhibiting identical signs, had undergone extensive work-up, and was nearing euthanasia. In this instance, the owner was alerted to the paper by her veterinarian and reached out. The veterinarian was understandably cautious about using phenytoin in a cat. Ultimately, we all connected, treatment was initiated, and the cat recovered. While this case report did not provide earth-shattering observations like Alois Alzheimer's report, it did positively impact the life of one cat, and that cannot be bad.

References:

1. Alzheimer, A. 1907 Uber eine eigenartige Erkrankung der Hirnrinde. Allgemeine Zeitschrift fur Psychiatrie und phy-chish-Gerichtliche Medizin, (Berlin) 64: 146-148.
2. Alzheimer, A., Stelzmann, R. A., Schnitzlein, H. N. & Murtagh, F. R. An english translation of alzheimer's 1907 paper, "über eine eigenartige erkankung der hirnrinde." Clin. Anat. 8, 429–431 (1995).
3. Bagley, R. S., Pluhar, G. E. & Alexander, J. E. Lateral intervertebral disk extrusion causing lameness in a dog. J Am Vet Med Assoc 205, 181–3; discussion 183-5 (1994).
4. Galano, H. R., Olby, N. J., Howard, J. F. & Shelton, G. D. Myokymia and neuromyotonia in a cat. J Am Vet Med Assoc 227, 1608–1612 (2005).

Martí Pumarola Batlle

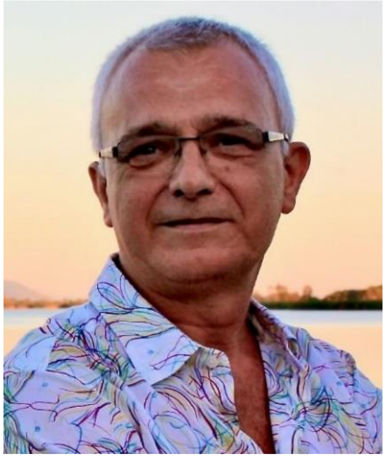

My experience in Veterinary Pathology over more than 40 years has led me to receive, study, and interpret numerous diseases affecting the nervous tissue of animals. Neuropathology has established itself as an essential branch that supports the development of veterinary neurology.

However, getting to where we are today has required considerable effort under very different conditions. Initially, most of the neurological case samples we received came from clinical veterinarians who, intrigued by a neurological case with an unclear diagnosis, wanted to go beyond clinical evaluation and imaging to resolve it. In recent years, with the presence of qualified veterinary neurologists, the quality of neurological diagnoses has greatly improved, the number of cases has increased, and new lesional patterns have been identified. All of this has led to increased interest in histopathological studies, contributing to the development of veterinary neuropathology.

Thanks to these advancements, we have identified new neuropathological forms that had not been previously described in veterinary medicine but had been recognized in humans. We have been able to develop veterinary neuropathology in a profound and diverse way, applying both basic and specialized techniques, including immunohistochemistry and molecular biology. Clinical and neuropathological correlation has proven to be essential, and our diagnoses have been strengthened by correlating them with those described in human medicine. Most importantly, these advancements have confirmed the potential of our pets as animal models for the study of many human diseases.

However, despite the wealth of information we received from neurological case samples, most of them were isolated cases. Despite the application of various diagnostic techniques, it has become increasingly difficult for the scientific community to access these findings. Given the reluctance of many scientific journals to publish isolated cases, and the difficulty or impossibility of obtaining similar cases, many of these studies have been stored away and, unfortunately, many have been lost to veterinary neuropathology.

I wholeheartedly support the publication of isolated cases, as long as they are well-referenced and accompanied by as much information from the various scientific fields as possible. I am convinced that this will greatly contribute to the development of veterinary neurology.

John H. Rossmeisl

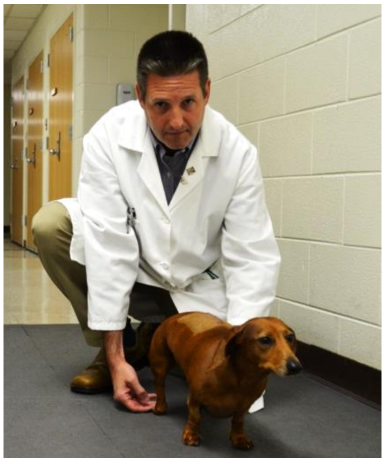

Case reports, in various forms, have existed for over 4,000 years and represent the oldest and most fundamental means by which practitioners of medicine record, describe, and disseminate information about a patient with a new disease, unusual features of a known condition, novel insights into a known disease, or an unexpected or undescribed outcome from an intervention (1). Although case reports occupy the lowest level in the evidence-based medicine hierarchy, they remain highly relevant to clinical practice, medical education, and biomedical research. Case reports provide a crucial platform for communicating unique clinical experiences, diagnostic or therapeutic innovations, and the formulation of new hypotheses—all of which contribute to the advancement of medical knowledge and patient-centered care. The structure of case reports also provides detailed insight into important aspects of daily medical practice, such as clinical decision-making processes.

For many students, early-career clinicians, or researchers, case reports represent their first effort to publish in the medical literature. The creation of a well-written case report is a useful exercise for developing knowledge integration, critical thinking, and scientific communication skills, and it introduces aspiring authors to the rigor and processes associated with peer review.

A case report that established the association between the Helicobacter pylori bacterium and Crohn's disease ultimately led to the receipt of the 2005 Nobel Prize, emphasizing the potential impact of case reports (2). In veterinary medicine in particular, case reports provide clinicians with valuable information when other evidence is often unavailable.

There are several notable limitations of case reports. Inherently, case reports are uncontrolled, observational studies of a single animal, making generalization and causal inference impossible. The retrospective nature of case reports makes them subject to recall bias, and there is a publication bias toward positive interventional case reports, with very few examples describing therapeutic failures (3). Modern publishing models and practices threaten case reports, as some editors and journals consider them detrimental to citation and other metrics that contribute to a publication's impact factor.

I believe the strengths of case reports outweigh the weaknesses associated with studying a single patient. By being aware of their limitations and adhering to recommended reporting guidelines, case reports can continue to contribute to the evolution of evidence-based medicine (4). The fact that case reports continue to be published in reputable medical journals further supports their relevance and importance to the global healthcare community.

References:

1. Nissen T, Wynn R. The history of the case report: a selective review. JRSM Open. 2014 Mar 12;5(4):2054270414523410. doi: 10.1177/2054270414523410.
2. Mégraud F. A humble bacterium sweeps this year's Nobel Prize. Cell. 2005 Dec 16;123(6):975-6. doi: 10.1016/j.cell.2005.11.032.

3. Albrecht J, Meves A, Bigby M. Case reports and case series from Lancet had significant impact on medical literature. J Clin Epidemiol. 2005 Dec;58(12):1227-32. doi: 10.1016/j.jclinepi.2005.04.003.
4. Riley DS, Barber MS, Kienle GS, Aronson JK, von Schoen-Angerer T, Tugwell P, Kiene H, Helfand M, Altman DG, Sox H, Werthmann PG, Moher D, Rison RA, Shamseer L, Koch CA, Sun GH, Hanaway P, Sudak NL, Kaszkin-Bettag M, Carpenter JE, Gagnier JJ. CARE guidelines for case reports: explanation and elaboration document. J Clin Epidemiol. 2017 Sep;89:218-235. doi: 10.1016/j.jclinepi.2017.04.026.

Koen M. Santifort

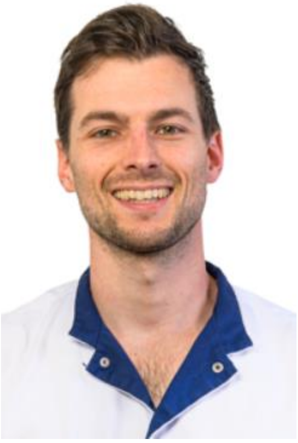

Case reports have strengths and limitations, scientifically speaking. However, in my opinion, the value of case reports outweighs their limitations in various ways. During the training phase, as a veterinary student or specialist-in-training, each case has unique aspects that can be recognized and appreciated.

When appreciated to their fullest extent, each case offers valuable learning opportunities. Even when a case does not present with unique aspects at first glance, it can be the mundane or even commonplace characteristics that reemphasize important clinical decision-making processes. It is the collective learning opportunities from these individual cases that form the backbone of every veterinarian's or specialist's career. It would be negligent to discount the value of single cases throughout one's career.

Case reports provide authors with the opportunity to document novel or unusual, clinically useful, and significant clinical signs, findings, treatments, or outcomes. Many case reports include "this is the first…" in their abstract, introduction, or discussion, highlighting their role in presenting new information. These sentences may be disliked by readers or reviewers for several reasons: 1) it can be difficult to know with certainty that a similar case has not been reported before (e.g., in non-digitalized publications), 2) the value of case reports should not be reduced to "being the first," and 3) over-emphasizing rarity may detract from the clinical usefulness or interest to readers.

When assessing the value of case reports, the low level of scientific (statistically significant) evidence that can be gained from case reports is often mentioned as a primary argument for judging them to have little value. Aside from providing definite proof of, for instance, possibilities (e.g., a good outcome in a case where a diagnosis was previously associated with only poor outcomes), case reports can be valuable in other ways:

- Some conditions are too rare for large-scale studies, making case reports the only way to document and share knowledge about them.
- Case reports often highlight unexplored areas, kindling larger studies like cohort studies or clinical trials. In this respect, they can form an essential first step in the research process.
- They may provide insights into unusual treatment responses, adverse effects, or complications.
- Case reports are a valuable teaching tool for those writing them and/or reading them. They are uniquely suited to offering real-world examples of diagnostic reasoning and management decision-making.
- Case reports offer the best available evidence in certain situations, such as when other formal study designs are not possible due to ethical, practical, or logistical considerations.
- New conditions may start out as rare but may (soon or later) become commonplace. Case reports serve as a 'warning' in that respect.

- Case reports may spark interest in a particular subject and provide readers with a broader insight into the various aspects of the case. The foundation of science is curiosity, the hunger for knowledge. A practical example of a relevant medical case can make all the difference in sparking readers' curiosity and interest.

It is these opportunities and characteristics of case reports that should be valued, not only their capacity to provide scientific or statistical proof.

Simone Spinillo

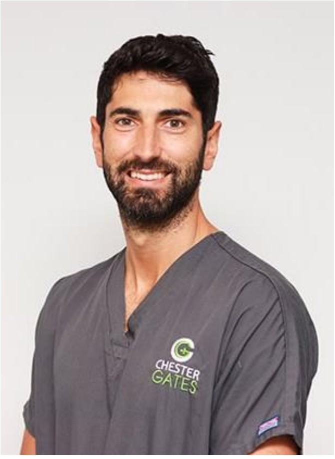

Veterinary medicine thrives thanks to the dedication of veterinarians who devote their time to preparing veterinary research and case reports. For many, publishing in a peer-reviewed journal may start as a hobby, but it has become an essential milestone for veterinary residents working toward their credentials. Often, a case report is the first step into the publishing world and is sometimes seen as a more accessible way to get an article accepted.

Case reports are invaluable as they provide detailed insights into the diagnosis, treatment, and prognosis of specific clinical cases. They often highlight new diseases, unusual presentations of known conditions, or new therapeutic options, all of which are incredibly beneficial for both clinical practice and research. One of the main challenges in veterinary literature is the limited number of cases researchers can gather, making case reports particularly important.

These reports offer a unique opportunity for professionals and students to immerse themselves in real clinical scenarios, following a case from start to finish. This hands-on experience is vital as it bridges the gap between theoretical knowledge and practical skills. Case reports present evidence-based solutions to complex or rare clinical cases, making them an indispensable resource for any veterinarian. Even when they focus on rare diseases or unique cases, they lay the groundwork for future hypotheses and more comprehensive studies. Additionally, case reports can promote interdisciplinary collaboration by highlighting cases that might interest professionals in related fields.

However, case reports do have their limitations. They often rely on single cases or small series, and unusual or positive outcomes are more likely to be reported. Despite these limitations, case reports are, in my view, more informative than educational, offering a wealth of practical knowledge and insights that are essential for the growth and development of veterinary medicine.

Brian A. Summers

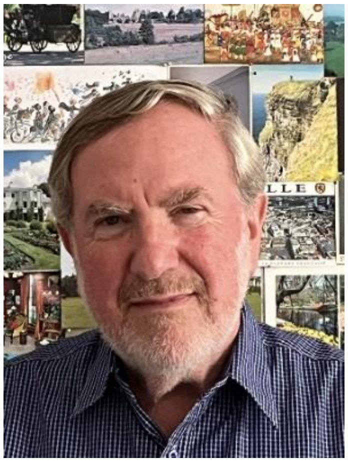

Case reports: a necessary if imperfect fact of life

Case reports from the medical, veterinary medical, and allied professions have long appeared in the journals of their associations and in some other publications. In 1817, surgeon James Parkinson contributed *An essay on the shaking palsy,* based on his experience with six adult male patients (1). More than two centuries later, this condition is known globally as Parkinson's disease; good case reports can have a massive and lasting impact.

In identifying a new disorder in such reports, the name chosen is a major consideration and when breaking new ground, there is merit in keeping it simple. The French neurologist Jean-Martin Charcot identified a debilitating, neuroinflammatory disorder of humans. In the English language, this condition subsequently became known as Multiple Sclerosis, which conveys that multifocally, the affected CNS white matter is firm to hard. Remarkably, this seemingly simplistic definition is universally recognized and endures. Very occasionally, names prove to be a mistake. Human synovial sarcomas were associated with various synovial structures but subsequently became identified widely, arising in the lung, mediastinum, gastrointestinal tract, abdominal wall, etc. Now, diagnosis is aided by immunocytochemistry and a tumor-specific chromosomal translocation. While the error is well known, such as a 1984 publication that recommends abandoning the term (2), the name persists.

As knowledge of biology and disease has expanded at an ever-increasing pace, the scope of case reports has changed dramatically. As in Parkinson's essay, the earliest offerings were largely based on patient history and repeated clinical examinations to document the deficits and the clinical course and tended to be lengthy. Today's case reports can employ a plethora of investigative tools - contemporary imaging (CT, MRI), PET scans, electrodiagnostic studies, PCR, in situ hybridization, molecular genetics and much more. Today, the first report of a new inherited disorder might name the responsible gene and even the nature of the mutation.

Arguably, when graduating with our various medical degrees, everything we have been taught draws on the observations and investigations of others who have gone before. This implies a sense of obligation for the next generation to substantiate and expand the pool of knowledge. Our earliest attempts at professional writing are often case reports – as novices, it's what we can do. While these can be based on single cases, if an option, a larger pool is a safer bet for recording a new syndrome accurately. To delay may be a choice for pathologists, whereas clinicians may feel compelled to record the first case of a new syndrome, to inform their colleagues without delay.

Case reports of deliberate physical injuries suffered by animals have been published, and they are comparable to those seen in battered children (3). Dogs and cats presented to veterinarians with such non-accidental injuries raise a warning that this violence in the household may extend beyond the family pets. These reports were instrumental in the evolution of forensic veterinary pathology.

The creation of newly named syndromes commonly evolves from multiple, earlier, case descriptions. One 1985 communication entitled *Unusual multisystemic vascular lesions in a cat* described a novel, feline, occlusive intravascular syndrome. Others reports followed (cited in reference 4), each adding a little flesh to the bones. By 2005, our Cornell group could add eight to the four cases already published and we proposed the name feline, systemic, reactive, angioendotheliomatosis (4). This diagnosis has been used in subsequent publications and in a further species. From this example, the necessity of the primary case reports is evident, setting the stage for something more definitive to evolve.

Finally, perhaps once in a lifetime, case reports present something radically different from anything in your prior experience. I refer to the leukoencephalomyelopathy of domestic cats fed dry cat food that has been gamma irradiated. The intent was to achieve sterility, such as in a germ-free laboratory setting, or for quarantine/importation purposes, but the outcome was disastrous. Our faculty were consulted on a laboratory-based nutritional study in which almost all cats developed ataxia - and we were clueless. At first a huge mystery, eventually the connection with the diet and specifically its prior treatment was recognized and published; this has been experimentally substantiated (5,6). Proposals as to the effects of irradiating the food have suggested a vitamin A deficiency or a fatty acid abnormality (5,7).

References**:**

1. Parkinson J 1817. An essay on the shaking palsy. Whittingham and Rowland for Sherwood, Needly and Jones, London
2. Miettinen M, Virtanen I. Synovial sarcoma--a misnomer. Am J Pathol. 1984 Oct;117(1):18-25.
3. Munro HM, Thrusfield MV. 'Battered pets': features that raise suspicion of non-accidental injury. J Small Anim Pract. 2001 May;42(5):218-26. doi: 10.1111/j.1748-5827.2001.tb02024.x.
4. Fuji RN, Patton KM, Steinbach TJ, Schulman FY, Bradley GA, Brown TT, Wilson EA, Summers BA. Feline systemic reactive angioendotheliomatosis: eight cases and literature review. Vet Pathol. 2005 Sep;42(5):608-17. doi: 10.1354/vp.42-5-608.
5. Cassidy JP, Caulfield C, Jones BR, Worrall S, Conlon L, Palmer AC, Kelly J. Leukoencephalomyelopathy in specific pathogen-free cats. Vet Pathol. 2007 Nov;44(6):912-6. doi: 10.1354/vp.44-6-912.
6. Caulfield CD, Kelly JP, Jones BR, Worrall S, Conlon L, Palmer AC, Cassidy JP. The experimental induction of leukoencephalomyelopathy in cats. Vet Pathol. 2009 Nov;46(6):1258-69. doi: 10.1354/vp.08-VP-0336-C-FL.
7. van den Ingh TSGAM, Grinwis GCM, Corbee RJ. Leukoencephalomyelopathy in cats linked to abnormal fatty acid composition of the white matter of the spinal cord and of irradiated dry cat food. J Anim Physiol Anim Nutr (Berl). 2019 Sep;103(5):1556-1563. doi: 10.1111/jpn.13139.
